# Role of imaging in rare COVID-19 vaccine multiorgan complications

**DOI:** 10.1186/s13244-022-01176-w

**Published:** 2022-03-14

**Authors:** Riccardo Cau, Cesare Mantini, Lorenzo Monti, Lorenzo Mannelli, Emanuele Di Dedda, Abdelkader Mahammedi, Refky Nicola, John Roubil, Jasjit S. Suri, Giulia Cerrone, Daniela Fanni, Gavino Faa, Alessandro Carriero, Angelo Scuteri, Marco Francone, Luca Saba

**Affiliations:** 1 Department of Radiology, Azienda Ospedaliero Universitaria (A.O.U.), di Cagliari – Polo di Monserrato, s.s. 554 Monserrato, 09045 Cagliari, Italy; 2 Department of Neuroscience, Imaging and Clinical Sciences, ‘G. d’Annunzio’ University, Chieti, Italy; 3 Department of Biomedical Sciences, Humanitas University, Via Rita Levi Montalcini 4, Pieve Emanuele, 20090 Milan, Italy; 4 IRCCS Humanitas Research Hospital, Via Manzoni 56, Rozzano, 20089 Milan, Italy; 5 Department of Radiology, IRCCS, SDN, Naples, Italy; 6 Department of Neuroradiology, University of Cincinnati Medical Center, Cincinnati, OH USA; 7 Roswell Park Cancer Institute, Jacobs School of Medicine and Biomedical Science, Buffalo, NY USA; 8 Stroke Diagnosis and Monitoring Division, Atheropoint LLC, Roseville, CA USA; 9 Department of Pathology, Azienda Ospedaliero Universitaria (A.O.U.) di Cagliari, Cagliari, Italy; 10 Department of Diagnostic and Interventional Radiology, AOU Ospedale Maggiore Della Carità Di Novara, Novara, Italy

**Keywords:** Covid-19, Vaccination, Adverse events

## Abstract

As of September 18th, 2021, global casualties due to COVID-19 infections approach 200 million, several COVID-19 vaccines have been authorized to prevent COVID-19 infection and help mitigate the spread of the virus. Despite the vast majority having safely received vaccination against SARS-COV-2, the rare complications following COVID-19 vaccination have often been life-threatening or fatal. The mechanisms underlying (multi) organ complications are associated with COVID-19, either through direct viral damage or from host immune response (i.e., cytokine storm). The purpose of this manuscript is to review the role of imaging in identifying and elucidating multiorgan complications following SARS-COV-2 vaccination—making clear that, in any case, they represent a minute fraction of those in the general population who have been vaccinated. The authors are both staunch supporters of COVID-19 vaccination and vaccinated themselves as well.

## Key points

- Different vaccines against SARS-COV-2 have been authorized in clinical practice.
- Post-vaccination COVID-19 adverse events have been described.
- Non-invasive imaging should be performed in patients with a clinical suspicion after vaccination.

## Introduction

COVID-19 is a pandemic with dramatic consequences for global health leading to high death rates [1, 2].

Different vaccines against SARS-COV-2 have been authorized in clinical practice to prevent the spread of the disease pandemic and reduce mortality [3]. The spike protein of SARS-COV-2 represents the most suitable target and various vaccines have been developed with different platforms, including viral vector vaccines and mRNA vaccines [3].

The most commonly described side effects after COVID-19 vaccination are pain at the injection site, fever, muscle pain, fatigue, and headache [4].

**Fig. 1:**
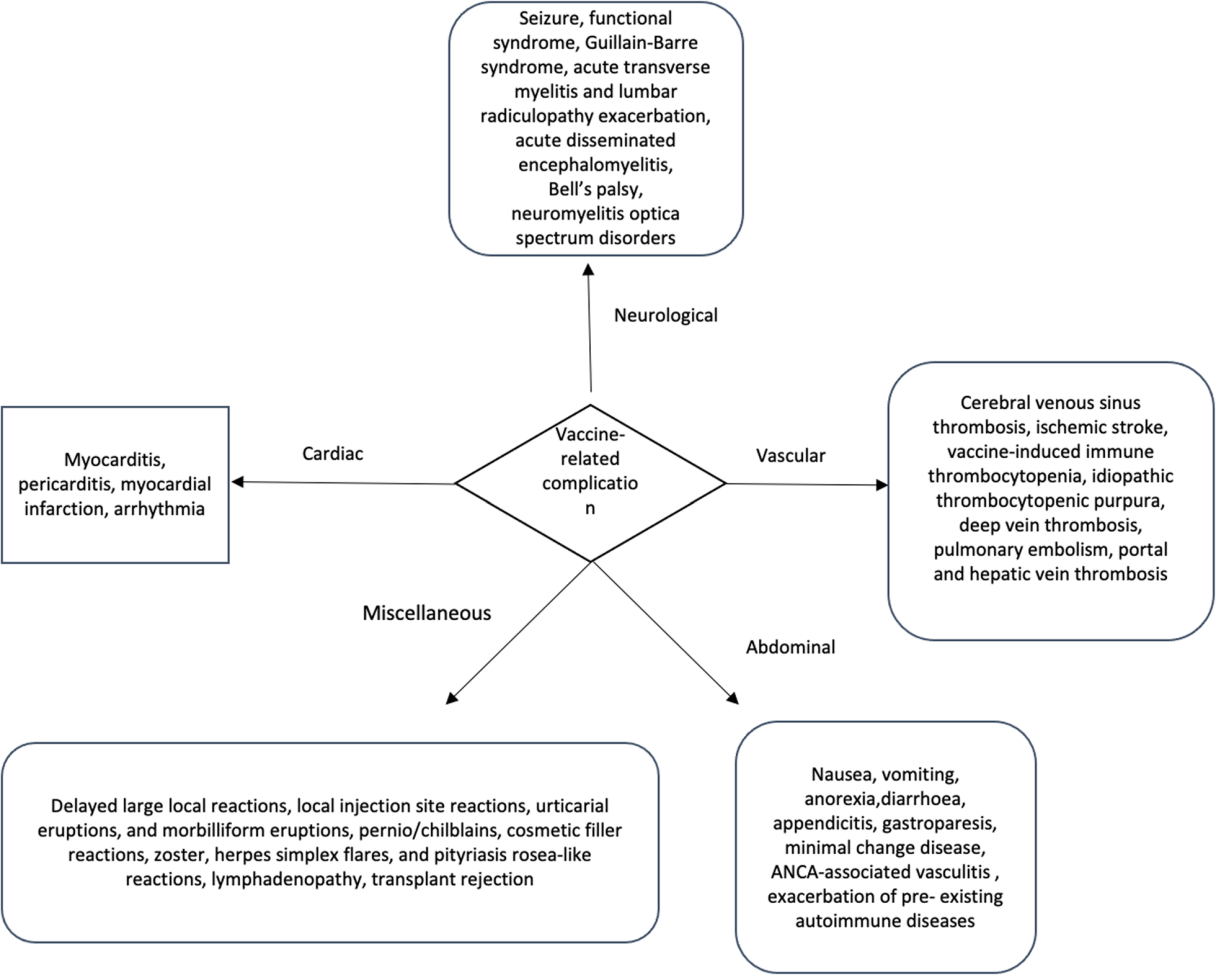
Vaccine-related side effects in different organs

An adverse event following vaccination is referred to as any unpleasant medical event after vaccination, without a definite causal relationship to the vaccine whether it’s either local or systemic [5]. Despite clinical trial data regarding safety and efficacy data of COVID-19 vaccines, multiple case reports, and case series have described rare but serious adverse events, with multiorgan involvement including brain, heart, and vascular system (Fig. 1) [6–15].

We present the complications reported following SARS-COV-2 vaccination and discuss both the adverse effects and their mechanisms in the current literature. This review also shed the light on the role of imaging for early detection of these potentially life-threatening complications. Even though we discuss the complications of the vaccine, the benefits outweigh the risk of being severely infected with SAR-COV-2.

## Cardiac complications

### Background

Vaccine-related cardiac complications have been reported as a rare adverse events after vaccination, especially Smallpox vaccination [16], Hepatitis B, Anthrax, and Haemophilus influenzae vaccination [17]. Myopericarditis was the most frequently described cardiac complication after vaccination. Before the COVID-19 pandemic, the Vaccine Adverse Event Reporting System (VAERS) reported 708 patients who met the diagnosis of myopericarditis among 620,195 individuals between 1990 and 2018, with a rate of 0.1% [17].

Eckart et al. evaluated 540,824 patients following Smallpox vaccination for vaccine-related myopericarditis. Among them, myocarditis was diagnosed in 67 patients, reporting an objective normalization of cardiac function at follow-up, and 20% of patients with persistent symptoms despite normal testing [18]. Given these findings, the authors suggested considering vaccine-related myocarditis in a patient with chest pain after vaccination [18].

As of August 4, 2021, a total of 4.27 billion doses of the COVID-19 vaccine were used, and thus far cardiac complications were reported all over the world during the first wave of COVID-19 vaccination.

Recently, the Center for Disease Control and Prevention (CDC) described a likely association between the mRNA vaccine and myocarditis and pericarditis, cataloged as “probable myocarditis”, “confirmed myocarditis”, and “acute pericarditis” [11]. CDC data suggested that myopericarditis was more common in young adults, male, and identified predominantly after the second vaccine dose, compared with the first [11].

Several mechanisms for post-vaccination myocarditis have been hypothesized: (1) mRNA vaccine can induce an aberrant non-specific innate response [11, 19] or (2) a molecular mimicry mechanism between the viral spike protein and cardiac protein [20, 21]; or (3) high antibody response may have been generated in a small group of subjects, leading to a hyperimmune response [21]; or (4) autoantibodies generation against several antigens with functional effects on cardiomyocytes in susceptible individuals after vaccination [11, 22].

**Fig. 2:**
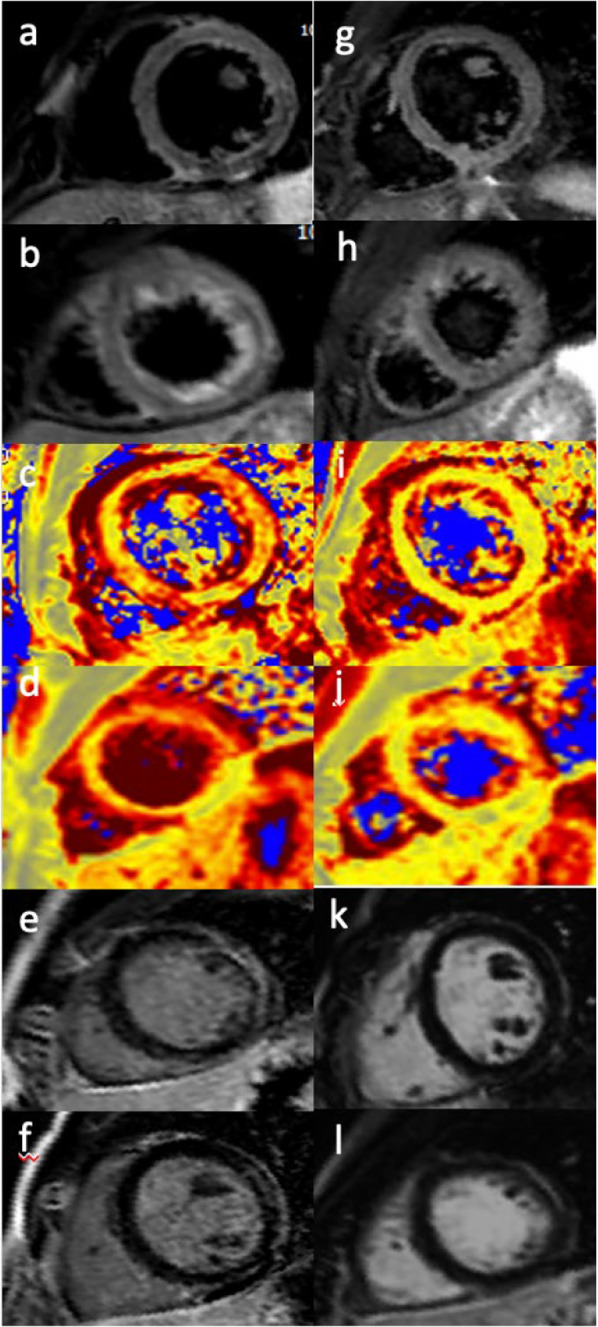
A 25-years-old-man with symptoms of fever, fatigue, shortness of breath and chest pain which developed one day following the second dose of COVID-19 vaccine. T2-short tau inversion recovery CMR short axis (**a**, **b**) demonstrating edema in the antero-lateral segments. T2 mapping short-axis view confirmed the presence of edema in the same segments (**c**, **d**). Late gadolinium enhancement short-axis view showed a subepicardial antero-lateral scar with an associated pericardial enhacement (**e**–**f**). Follow-up CMR was done 3 months from start of symptoms revealing the disappearance of edema in both the T2-short tau inversion recovery CMR short axis (**g**, **h**) and T2 mapping short-axis view (**i**, **j**). There is no evidence of focal areas of late gadolinium enhancement in the antero-lateral segments (**k**, **l**)

The Medicines and Healthcare Products Regulatory Agency (MHRA) also described some cases of myocarditis and pericarditis with the viral vector vaccine (i.e. AstraZeneca) [23, 24]. Similar cases were also reported in the EudraVigilance database [25]. Through July 21st, 2021, the MHRA also reported rate and rhythm disorder as well as myocardial infarction. In total, there were 181 recorded deaths following a vaccine-related cardiac complication [24]. Several papers described myocarditis and pericarditis following vaccine as self-limited and transient conditions [13, 26, 27]. Notwithstanding only long-term follow-up can reveal with certainty the permanent impact of this cardiac injury. See Fig. 2

A review of the worldwide literature reveals some cases of myocardial infarction either after mRNA vaccines and after viral vector vaccines [23, 25, 28–31].

Tajstra et al. described a clinical case of an 86-years old-man with acute ST-segment elevation myocardial infarction (STEMI) around 30 min after the injection of the first dose of Pfizer–BioNTech vaccine [31]. Similar findings were also reported with the viral vector vaccine [23].

The possible link between acute coronary syndrome and vaccination is unclear. Some authors speculated different theories, such as (1) a vasospastic acute coronary syndrome, namely Kounis syndrome, that involves mast cell activation through the release of inflammatory cytokines, leading to coronary artery vasospasm or atherosclerotic plaque rupture [31]; (2) post-vaccine physiological stress can destabilization of chronic atherosclerotic plaque; or (3) an immunologic response triggering of plaque rupture [29].

Nevertheless, there is still a lack of large multicenter studies and little evidence to establish a direct correlation between myocardial injuries and COVID-19 vaccines. To overcome this gap and evaluate the prevalence of myocardial damage after mRNA vaccines, a prospective study has been implemented [32].

Table 1 summarized previous research regarding vaccine-related cardiac complications.

**Table 1:** Previous case-report about vaccine-related cardiac complication

Authors	Cardiac complication	Type of vaccine	Number of patients described
Tajstra et al. [31]	Myocardial infarction	mRNA vaccine	1
﻿Muthukumar et al. [22]	Myocardial infarction	mRNA vaccine	1
﻿Srinivasan et al. [29]	Myocardial infarction	mRNA and viral vector vaccine	3
Chamling et al. [25]	Myocarditis	mRNA and viral vector vaccine	3
Sung et al. [30]	Myocardial infarction	mRNA vaccine	2
Abou et al. [28]	Myocardial infarction	viral vector vaccine	1
Isaak et al. [38]	Myocarditis	mRNA vaccine	1
Montgomery et al. [12]	Myocarditis	mRNA vaccine	23
Kim et al. [13]	myocarditis	mRNA vaccine	7
Marshall et al. [14]	Myocarditis	mRNA vaccine	7
Starekova et al. [15]	Myocarditis	mRNA vaccine	5

### Imaging

Clinicians should suspect vaccine-related myocarditis or pericarditis in patients with chest pain and a rise in cardiac enzyme. The diagnostic gold standard for the diagnosis of myocarditis is endomyocardial biopsy [33], however this procedure is infrequently used in clinical practice due to its invasive nature and limitations (e.g. sampling errors caused by focal or patchy involvement of myocardium, variability histopathological interpretation) [34]. Due to this uncertainty, several non-invasive imaging modalities help to diagnose myocarditis [35–37]. Beyond the first-line echocardiography, cardiac magnetic resonance (CMR) has emerged as a key tool in the diagnosis of myocarditis [37]. CMR features of vaccine-related myocarditis are similar to other virus-associated with myocarditis [25, 38].

Based on the Lake Louis criteria, CMR can identify myocardial damage with a diagnostic accuracy of 78% [39]. In addition, adding the parametric mapping techniques, such as T1 mapping, T2 mapping, and ECV to the classic CMR protocol may improve its accuracy, provide additional disease characterization, and help the management of different cardiac injuries [40].

**Fig. 3:**
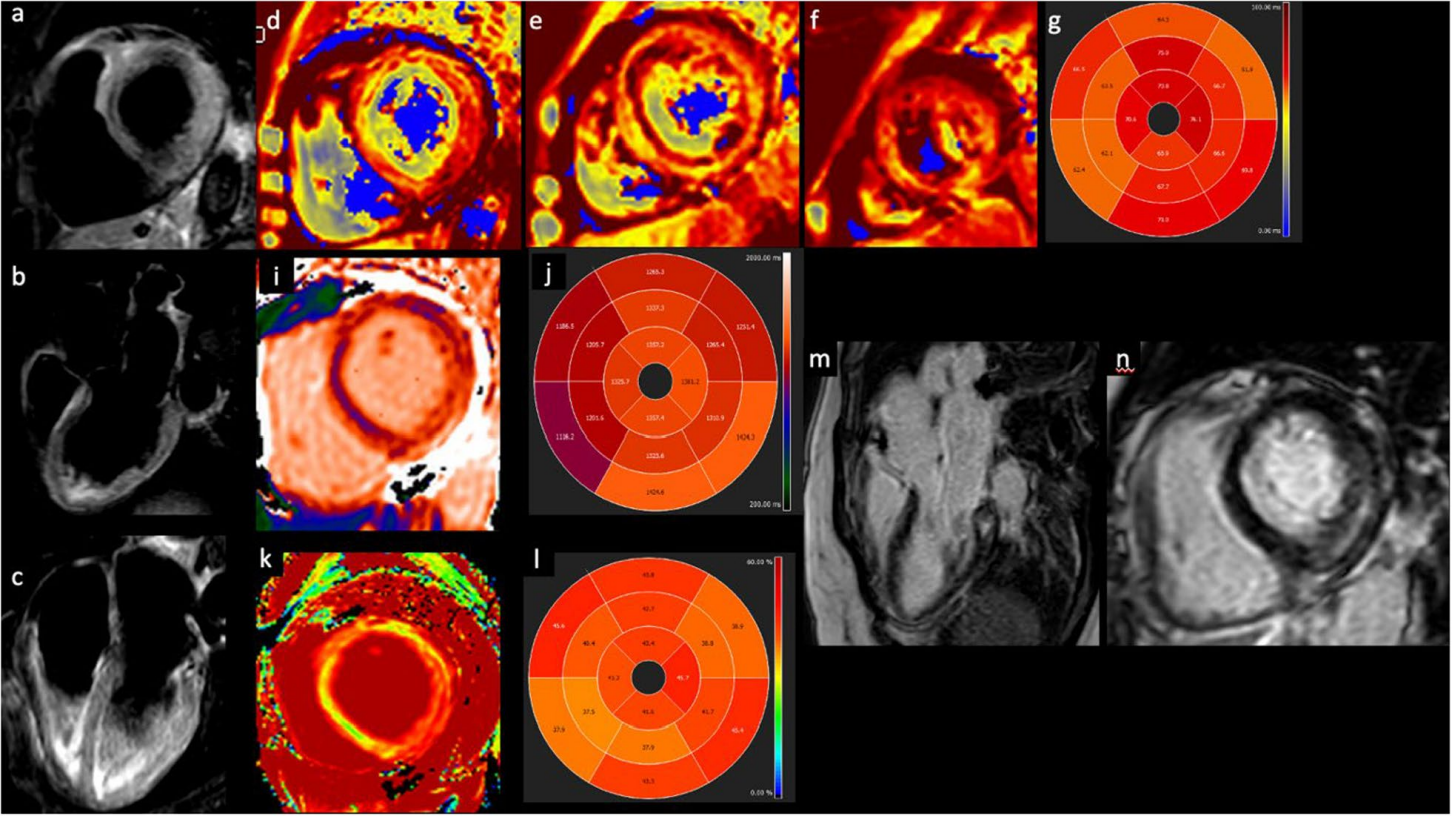
Myocarditis after COVID-19 vaccination. T2-short tau inversion recovery CMR short axis (**a**), three-chamber (**b**) and four-chamber view revealing edema in the infero-lateral basal segment. T2 mapping short-axis view and T2 mapping in AHA 16-segment model (**d**–**g**) showed altered values in all myocardial segments, especially in the infero-lateral basal segments. T1 mapping (**i**, **j**) and ECV (**k**, **l**) short-axis view confirming the altered values. Late gadolinium enhancement three chamber (**m**) and short-axis (**n**) view demonstrating an intramyocardial infero-lateral scar

Figure 3 demonstrated an example of CMR in patients with vaccine-related myocarditis.

There has been an association such other cardiac side effects, including myocardial infarction and arrhythmias [41].

Similarly, myocardial infarction should be suspected among patients presenting with acute chest pain to the emergency department after the COVID-19 vaccination. For an initial evaluation, ECG and cardiac troponin levels should be obtained. In accordance with 2020 ESC guidelines for the management of acute coronary syndrome, coronary computed tomography angiography (CCTA) may be an option in patients with low-to-intermediate clinical likelihood of acute coronary syndrome thanks to its negative predictive value to exclude coronary artery disease [42].

**Fig. 4:**
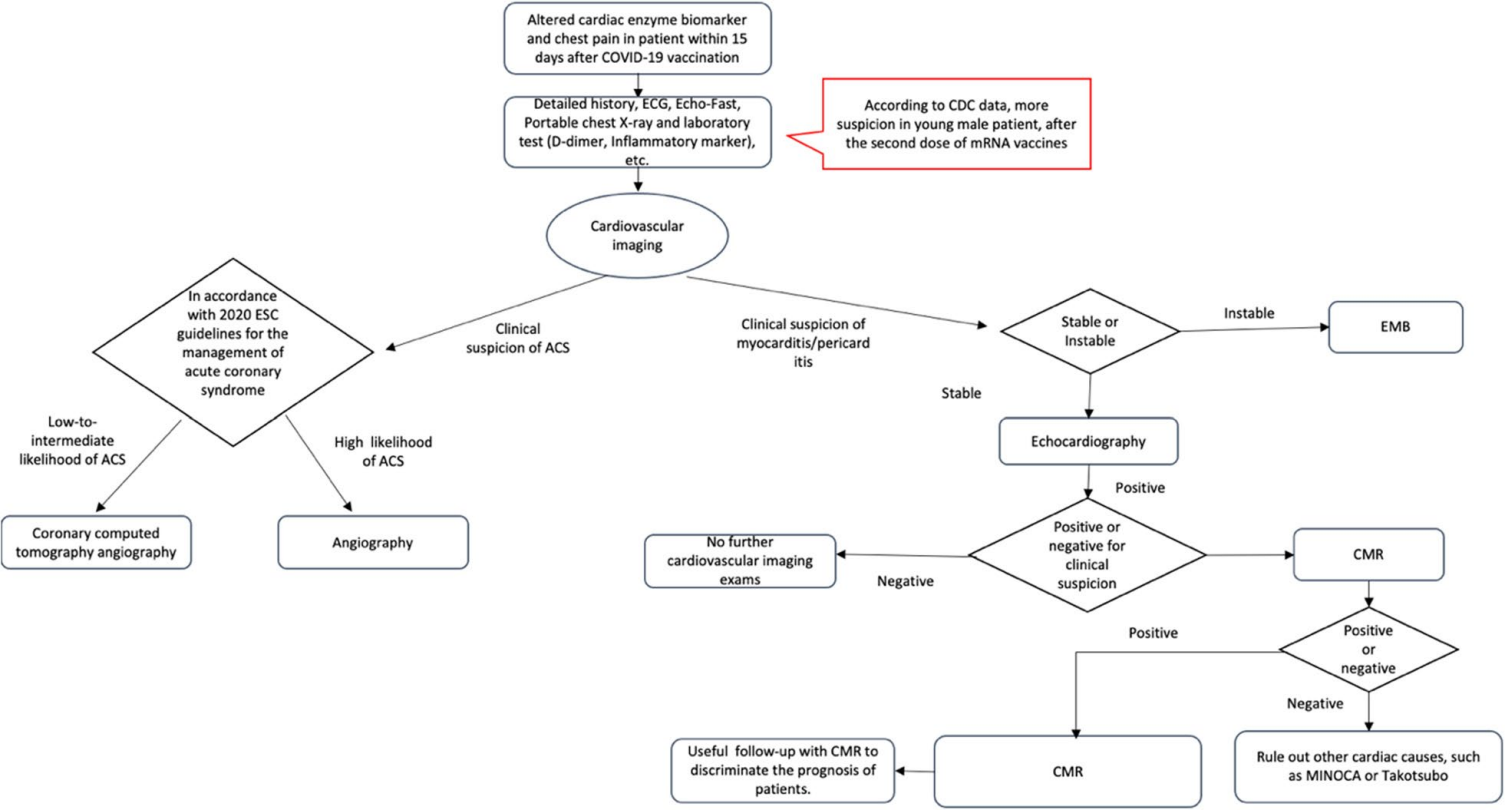
Diagnostic flowchart for suspected myocardial damage post COVID-19 vaccination

As reported in the previous studies [29, 30], imaging features are suggestive of acute thrombotic events as the underlying mechanism. CCTA can easily detect the presence of an intracoronary filling defect, while also evaluating the status of an atherosclerosis plaque. Thus excluding the presence of a vulnerable plaque as a trigger for thrombus formation [43]. Figure 3 showed a proposed diagnostic flowchart for suspected myocardial damage post-COVID-19 vaccination (Fig. 4).

## Neurological complications

### Background

Neurological disorders after COVID-19 infection are well known, with a spectrum of pathologies ranging from mild to severe [44]. Neurological complications after COVID-19 vaccination have emerged at the end of 2020 after 2 patients developed transverse myelitis following viral vector vaccination [45]. To date, out of 9,442 adverse events following immunization reported in the VAERS data related to mRNA vaccines, 254 (2.69%) were neurological [10]. A nationwide descriptive study by García-Grimshaw et al. including data from 704,004 first-dose recipients reported 33 (4.7/100,000 doses) serious adverse events. Out of these, 17 (51.7%; 2.4/100,000 doses) were neurologic complications, including seizure (0.99/100,000 doses), functional syndrome (0.56/100,000 doses), Guillain–Barre syndrome (0.43/100,000 doses), acute transverse myelitis (0.28/100,000 doses), and lumbar radiculopathy exacerbation (0.14/100,000 doses) [46]. On the other hand, the overall incidence of non-serious neurologic events was 600.7 cases per 100,000 administered doses [46]. Mild neurological side effects reported were headache (62.2%; 577.7/100,000 doses), transitory sensory symptoms (3.5%; 32.9/100,000 doses) and weakness (1%; 9.1/100,000 doses).

According to a trial with the Sinovac and Sinopharm vaccine, the most common neurological side effect after vaccination was headache (68%), and myalgia (60%) [47]. Among the 9442 reports of adverse events, the VAERS described also cases of stroke (17 cases), Guillain–Barre syndrome (32 cases), Bell’s palsy (190 cases), transverse myelitis (9 cases), and acute disseminated encephalomyelitis (6 cases) [48].

A frequently reported neurological side effect was Bell’s palsy [48–50]. Reports from the mRNA vaccine trials described 7 cases of 37,000 vaccine recipients who developed Bell’s palsy [48].

One explanation for this phenomenon is transient lymphopenia due to Type I interferons action following vaccination [50].

Neuroimmune complications were also described in some clinical cases, including Guillain–Barre Syndrome, ﻿Neuromyelitis Optica Spectrum Disorders, and Traverse Myelitis [9, 10, 51–53]. The development of a post-vaccination neuroimmune syndrome may be related to overactivation of the immune system after vaccination or a cross-reaction between host antibodies and proteins present in the peripheral myelin [51, 52]. Subsequently, the Guillen-Barre syndrome and ﻿Chronic Inflammatory Demyelinating Polyneuropathy Foundation suggested that individuals that developed Guillen-Barre Syndrome after their first immunization should avoid the second dose [54].

A clinical case by Vogrig et al. described a 56-year old female patient who developed an acute disseminated encephalomyelitis after the first dose of mRNA COVID-19 vaccine [55]. Similar cases were also reported by the VAERS [54] and by a case report out of China [56].

Table 2 reported previous research regarding vaccine-related neurological complications.

**Table 2:** Previous case-report about vaccine-related neurological complications

Authors	Neurological complications	Type of vaccine	Number of patients described
Allen et al	Guillan-Barre Syndrome	Viral vector vaccine	4
Maramattom et al	Guillan-Barre Syndrome	﻿Viral vector vaccine	7
Waheed et al	Guillan-Barre Syndrome	mRNA	1
García-Grimshaw et al	Adverse neurological events following vaccination	mRNA	6503
Chen et al	Neuromyelitis optica spectrum disorders	Viral vector vaccine	1
Malhotra et al	Trasverse myelitis	Viral vector vaccine	2
Roman et al	Trasverse myelitis	Viral vector vaccine	3
Vogrig et al	Acute disseminated encephalomyelitis	Viral vector vaccine	1
Cirillo et al	Bell’s palsy	mRNA vaccine and viral vector vaccine	
Soeiro et al	Bell’s palsy	mRNA vaccine	9
Mehta et al	Cerebral venous sinus thrombosis	Viral vector vaccine	2
Cao et al	Acute disseminated encephalomyelitis	Viral vector vaccine	1
Mayhani et al	Ischemic stroke	Viral vector vaccine	3
Blauenfeldt et al	Ischemic stroke	Viral vector vaccine	1
Suresh et al	Cerebral venous sinus thrombosis	viral vector vaccine	1

Recommendations suggest that comprehensive surveillance systems be in place to ensure vaccine safety and that the benefit of vaccination overcomes the risks [54].

### Imaging

Beyond the neuro-vascular adverse events following COVID-19 vaccination described in the following paragraph, other neurological adverse events were reported and should be managed with appropriate neuroimaging exams [9, 10, 54]. CT and MRI are the most important imaging techniques in the diagnosis of neurological disease. Most patients with neurological side effects related to the COVID-19 vaccination undergo neuroimaging and no specific findings are revealed [10, 53].

Guillain-Barré syndrome may occur following COVID-19 immunization. The classic neuroimaging pattern of this syndrome is characterized by cord T2 signal alteration and gadolinium enhancement of the caudal nerves roots. Waheed et al. reported a case of an 82-year-old highly functional female without significant comorbidities, with a suspected Guillain–Barre syndrome after mRNA vaccine, with typical MRI features of Guillain Barre syndrome [57].

Chen et al. described a patient who developed ﻿Neuromyelitis Optica Spectrum Disorder after vaccination for COVID-19, highlighting the usefulness of MRI in revealed area postrema and bilateral hypothalamus lesions without optic nerve and cervical spinal cord sparing [51].

The spectrum of neuroimaging abnormalities includes changes related to Guillain–Barre Syndrome, Bell’s palsy, Transverse Myelitis, Neuromyelitis Optica Spectrum Disorder, and acute disseminated encephalomyelitis [48, 54].

**Fig. 5:**
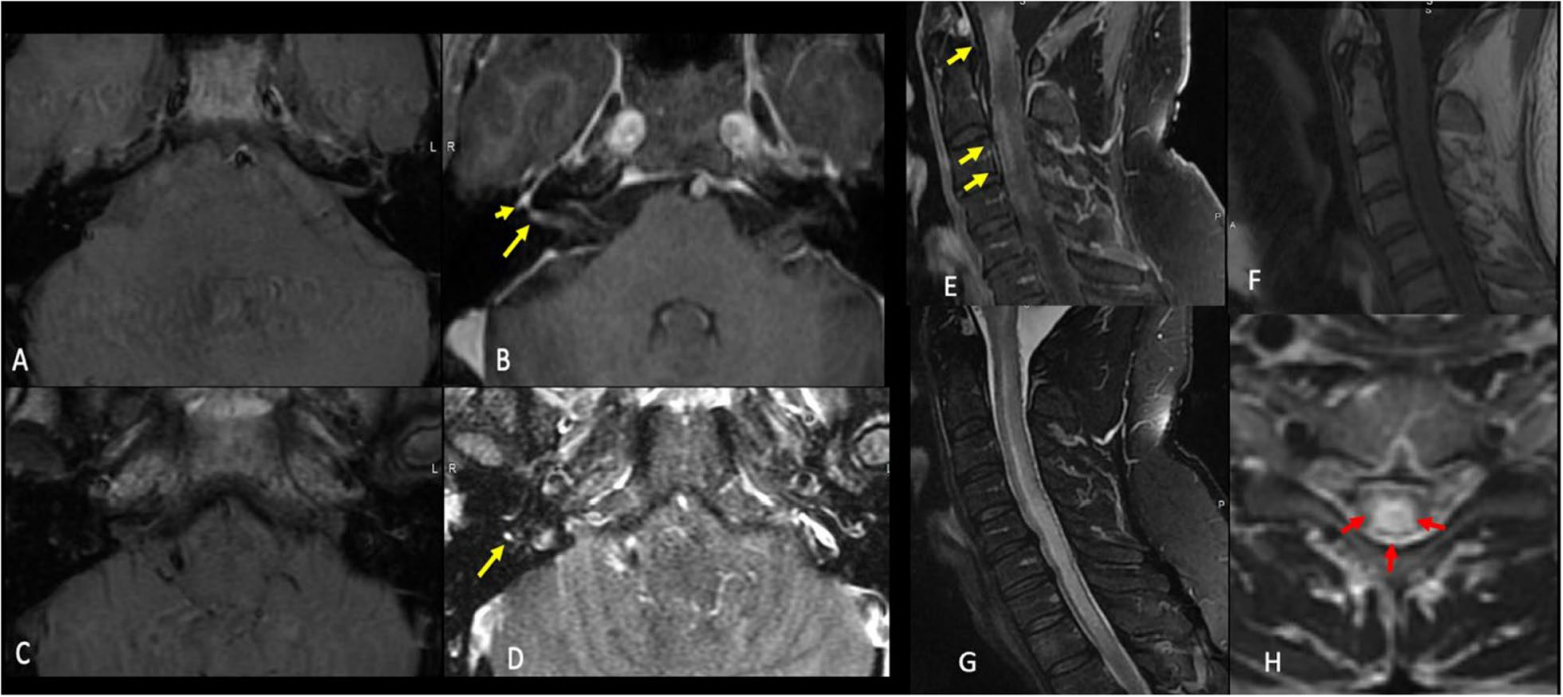
Neuroimaging features after COVID-19 vaccination. Case 1: A 46-year-old-man who presented with a rapid onset right-sided facial weakness after having COVID-19 vaccine. Axial T1 pre (**a** and **c**) and T1 postcontrast fat sat (**b** and **d**) demonstrate abnormal enhancement of the right facial nerve within the lateral right IAC (long yellow arrow in **b**) as well as asymmetric enhancement of the right geniculate ganglion (short yellow arrow in **b**) and tympanic portion of the facial nerve (yellow arrow in **d**). Findings are consistent with Bell's palsy. Case 2: A 51-year-old-man who presented with a sudden upper and lower limb weakness after having COVID-19 vaccine. Sagittal T1 postcontrast (**e**) T1 pre (**f**), STIR (**g**), and axial T2W (**h**) images demonstrate extensive T2 signal hyperintensity of the central cervical cord (red arrows) with patchy areas of enhancement at the levels of c1, c2, c3 and c4 (yellow arrows). There was no associated restricted diffusion. Findings were consistent with transverse myelitis

Figure 5 showed neuroimaging features following COVID-19 vaccination.

## Vascular complications

### Background

Recently, coagulopathy has been described after COVID-19 vaccination, especially following viral vector vaccine [58].

Mehta et al. reported two cases of cerebral venous sinus thrombosis after viral vector vaccine and proposed a potential immunological disorder supported by the presence of antibodies to platelet factor-4, with a mechanism similar to spontaneous heparin-induced thrombocytopenia. The authors suggest radiologists and neurologists be aware of this neurological complication after vaccination, particularly when comparing its management to traditional cerebral venous sinus thrombosis [59].

For an initial evaluation, beyond laboratory testing of blood count, non-contrast CT followed by CT venogram or magnetic resonance venography in selected patients is required [59].

Similar neurological complications were reported by the European Medicine Agency and by the MHRA [59]. The spectrum of vaccine-related coagulopathy was also shown by Al-Mayhani et al. [60], reporting three cases of ischemic stroke with large vessel occlusion after COVID-19 vaccination. Their observations suggest that immune-mediated coagulopathy, in addition to venous thrombosis, can involve arterial occlusion [60].

In view of several reports, the definition of a new syndrome has been proposed, namely vaccine-induced immune thrombocytopenia [59–66]. Additional cases have been also described for the mRNA vaccine by the European Medicine Agency, including at least 40 possible cases among 58 million recipients of the mRNA vaccine [67]. A recent autopsy report by Fanni et al. described a case of vaccine-induced immune thrombocytopenia in a 58-years old man 13 days after his first dose of the viral vector vaccine [61]. The report confirmed multiple microthrombi in unusual sites, including the heart, aortic vasa vasorum, lung, liver, kidney, and choroid plexus [61]. There have also been reports of post mRNA vaccination exacerbation of chronic idiopathic or immune thrombocytopenic purpura [68, 69]. The Scottish National Population-Based Database of 2.53 millions vaccinated individuals revealed a potential association between the viral vector vaccine and idiopathic thrombocytopenic purpura, with an incidence of 1.13 cases per 100,000 vaccinations [68].

Table 3 summarizes previous research regarding vaccine-related vascular complications.

**Table 3:** Previous case-report about vaccine-related vascular complications

Authors	Vascular complications	Type of vaccine	Number of patients described
Fanni et al	Thrombotic Thrombocytopenia	Viral vector vaccine	1
Schuktz et al	Thrombotic Thrombocytopenia	﻿Viral vector vaccine	5
Greinacher et al	Thrombotic Thrombocytopenia	Viral vector vaccine	11
Wolf et al	Thrombotic Thrombocytopenia	Viral vector vaccine	3
Toom et al	Exacerbation of familiar thrombocytopenia	mRNA	1
Blauenfeldt et al	Thrombocytopenia	Viral vector vaccine	1
Suresh et al	Thrombotic Thrombocytopenia	viral vector vaccine	1

### Imaging

Several international consensus guidelines about vaccine-induced immune thrombocytopenia diagnosis and management have been proposed, These are based upon laboratory and clinical findings first described in case reports of vaccine-induced immune thrombocytopenia [66, 70–72].

**Fig. 6:**
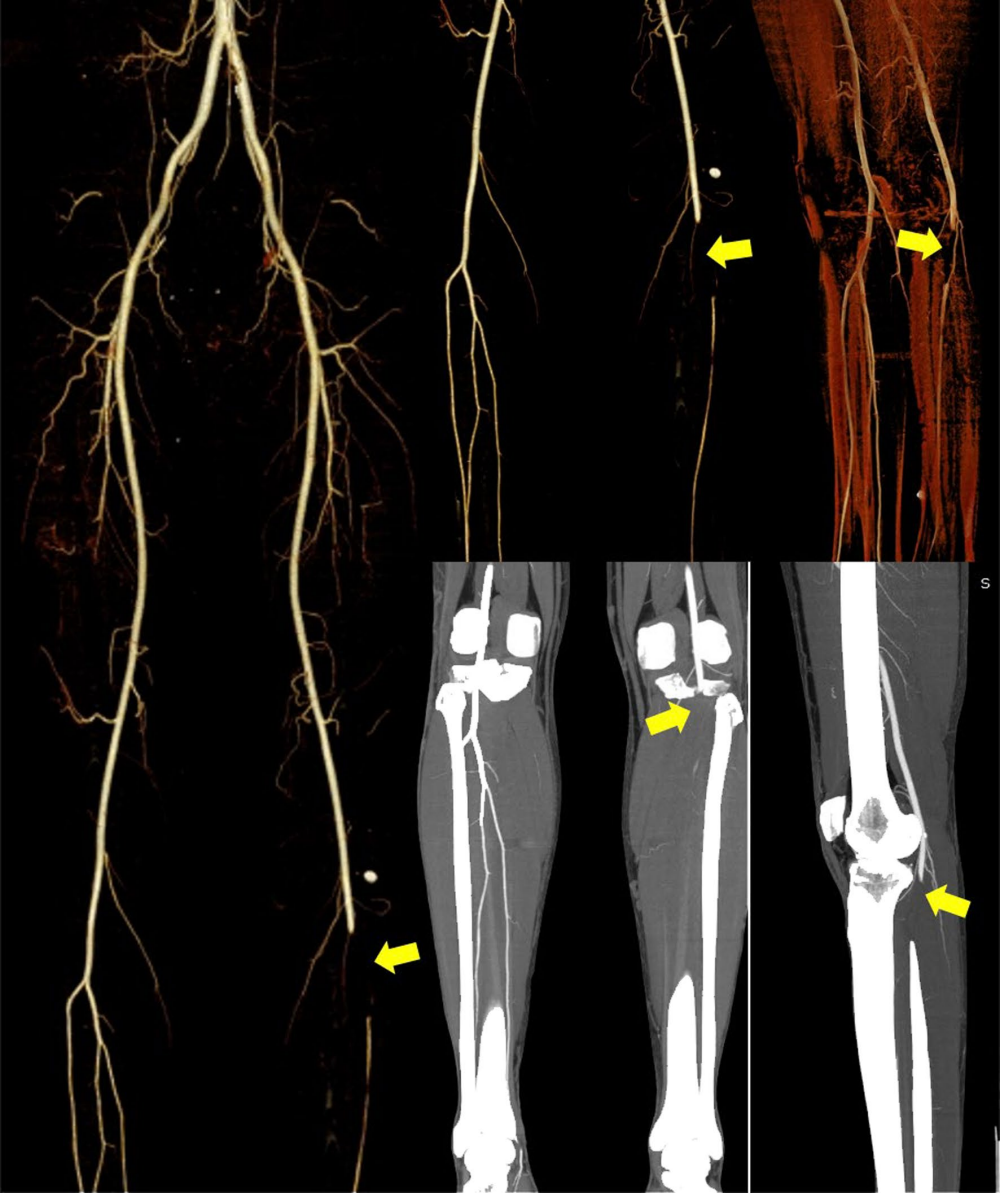
A 21-year-old, normal weight and non-smoker man presented to the emergency department with new onset of cool and painful left leg, ten days after having the second dose of COVID-19 vaccine from AstraZeneca. Volume Rendering and Maximum Intensity Projection CT angiography images show acute segmental thrombotic occlusion of the popliteal artery (yellow arrows). He had no underlying illnesses, trauma, surgery, infection, or immobilization. There was no known thrombophilia. Unfortunately, the patient underwent critical ischemia and urgent limb amputation

Based on these consensus guidelines, the patients would be classified as “definite case,” “probable case,” “suspected case,” and “unlikely case”. For patients with a high clinical suspicion of vaccine-induced immune thrombocytopenia, the guidelines suggest ordering imaging studied based on the location of symptoms to confirm the site of thrombosis, keeping in mind that VITT is associated with both arterial and venous thrombosis in a variety of sites [6, 71, 72]. Modern CT scans, thanks to constantly advancing technology with an increasingly better spatial and temporal resolution, can provide optimal and rapid imaging of the vessel lumen [73, 74]. In addition, CT scan is able to rule out organ complications, including mesenteric ischemia, infarcted bowel, and solid organ infarcts [75]. Even ultrasound represents a suitable imaging analysis, allowing an assessment of abdominal, lower, and upper limb vessels [72]. (See Fig. 6).Finally, MRI is an accurate alternative non-invasive imaging that can be performed in patients with neurological symptoms when evaluating for cerebral venous thrombosis [72, 75].

**Fig. 7:**
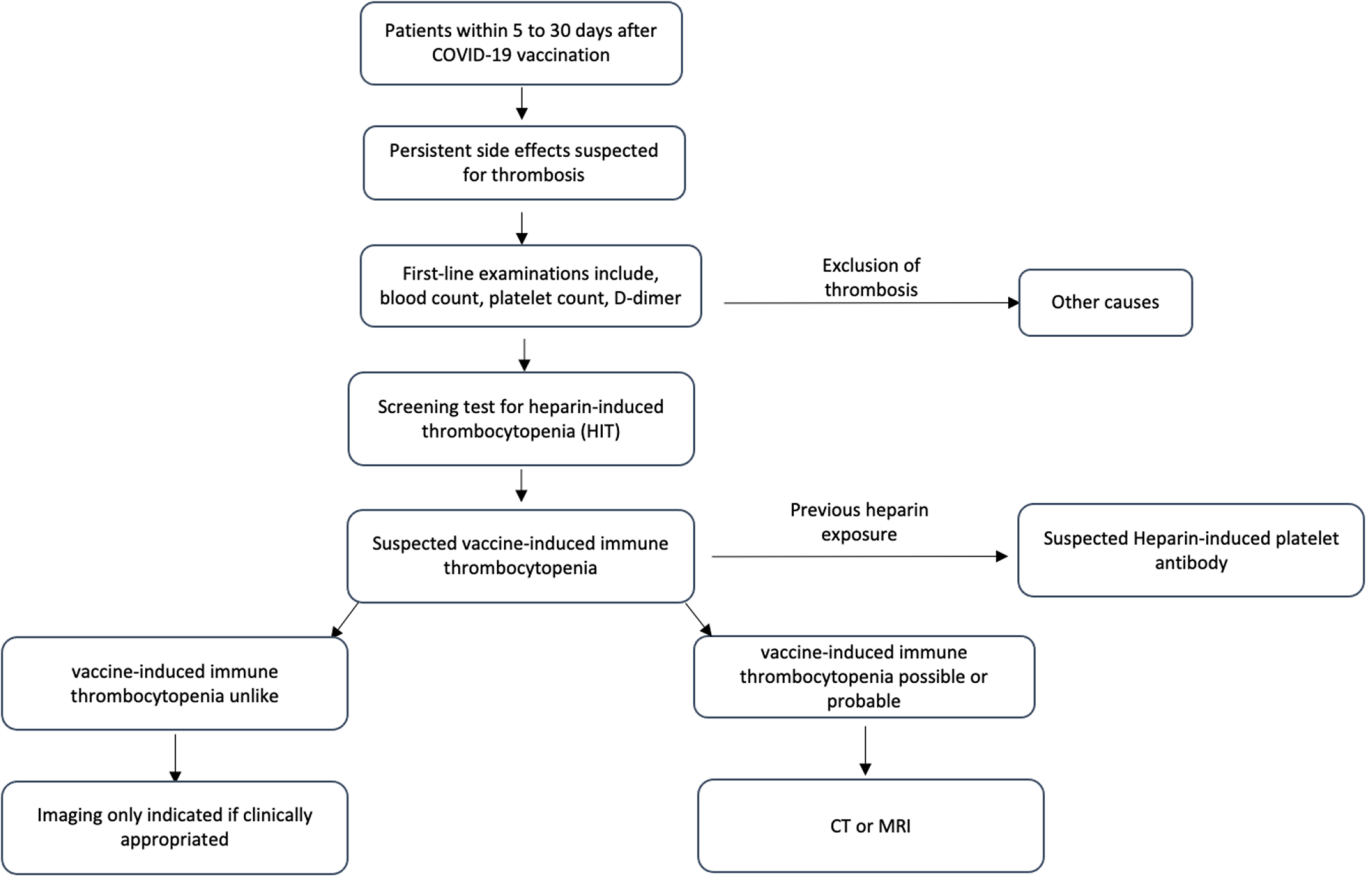
Diagnostic flowchart for suspected vaccine-induced immune thrombocytopenia

A diagnostic flowchart for suspected vaccine-induced immune thrombocytopenia was proposed in Fig. 7

Therefore, emergency clinicians should know this vascular complication when evaluating and managing patients after the COVID-19 vaccination, allowing a prompt diagnosis through non-invasive imaging to improve the patient outcomes.

**Fig. 8:**
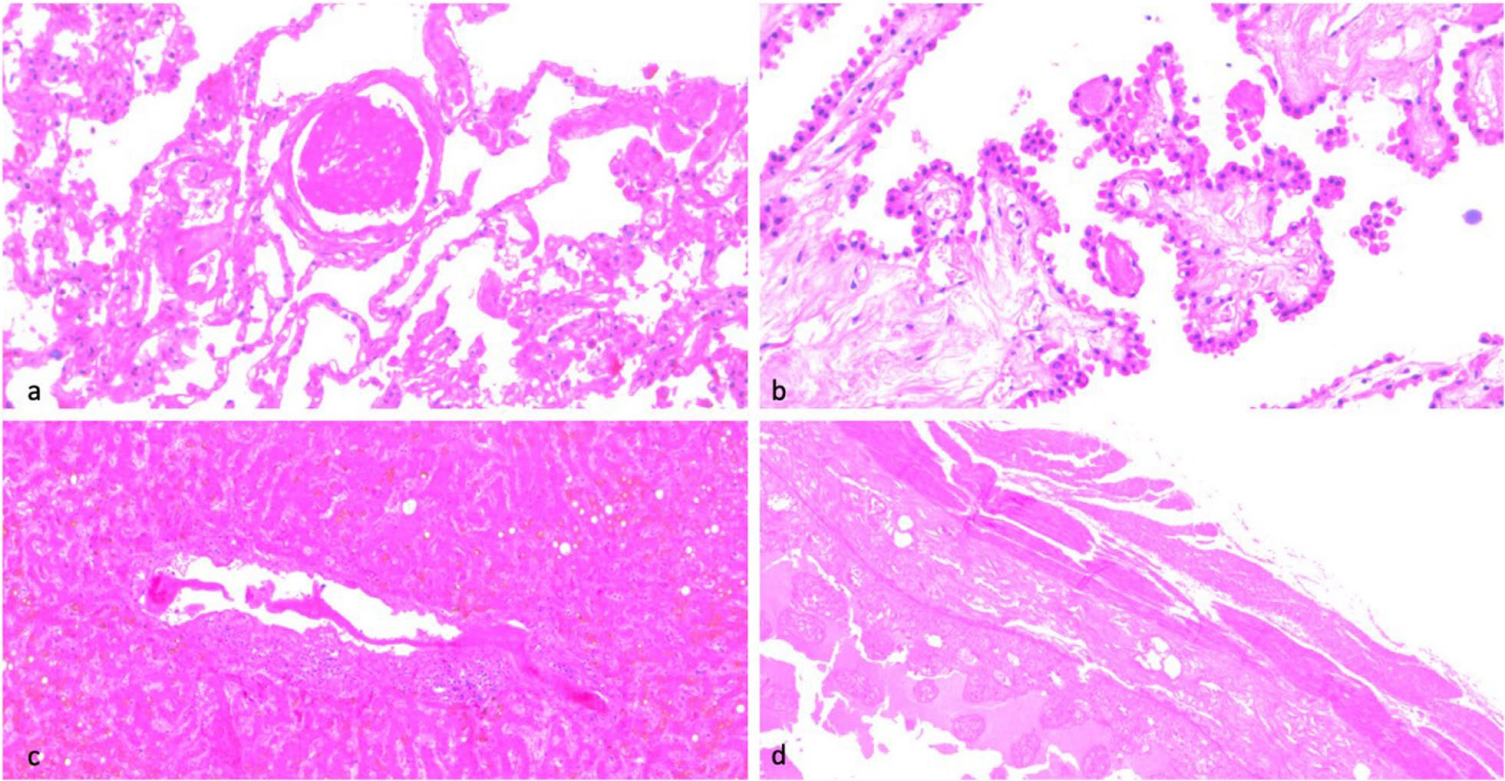
Histological sample of a patient who developed vaccine-induced immune thrombocytopenia after viral vector vaccine. **a** Lung. Thrombosis of a small arterial branch. Original magnification ×200. **b** Choroid plexuses. Multiple small thrombi are present inside the capillaries of choroid plexuses. H&E. Original magnification × 200. **c** Liver. Thrombosis of a portal vein branch. ×100. **d** Ileum. Diffuse severe hemorrhagic necrosis of the wall. ×50

Figure 8 showed a histological sample in a patient who developed vaccine-induced immune thrombocytopenia after viral vector vaccine.

## Abdominal complications

### Background

A high frequency of non-serious gastrointestinal adverse events was reported including nausea and vomiting, beyond these transitory post-vaccination side effects, some serious adverse events were described [24, 76, 77]. Of the more serious adverse events reported following vaccination, the most common was appendicitis, which was more frequent in younger populations [78]. These findings are in line with the CDC data, reporting appendicitis as the most common severe adverse event in the vaccine group compared with the placebo group [5]. A report by Scott et al. described a potential association between gastroparesis and mRNA vaccine in a previously healthy 57-years- old-man, that developed after both vaccine doses with refractory nausea and vomiting. A nuclear imaging study showed delayed gastric emptying, which improved after a course of prednisone. The authors concluded that the mechanism of action of mRNA vaccine may precipitate immune-mediated gastroparesis [79]. Several reports speculated that there may be a connection between COVID-19 vaccines and pre-existing autoimmune disease exacerbations [80–82]. COVID-19 vaccination has also been associated with the occurrence of glomerular diseases, including minimal change disease [83, 84]. Some authors formulated a hypothesis on how the COVID-19 vaccination can trigger glomerulonephritis, not implicating a direct action by the vaccine itself but rather a T-cell activation leading to podocyte injuries, or through molecular mimicry [84]. Other rare renal adverse events after vaccination against SARS-COV-2 were described as well. In particular, Shakoor reported a new onset renal limited ANCA-associated vasculitis in a 78- year- old woman with previously normal kidney function after receiving the mRNA vaccine [85]. Observational studies formulated a link between different infections and the development of vasculitis, with a poorly understood pathogenesis, that could involve a molecular mimicry mechanism between microbial peptides and antigens [86]. While there have been no reported cases of hepatitis in the registration trials [5, 24, 76], several reports have described biopsy proved autoimmune hepatitis in previously healthy patients [87, 88]. In a recent in-vitro study, a high affinity between antibodies against the spike protein S1 of SARS-COV-2 and human tissue proteins was reported—this included transglutaminase 3, transglutaminase 2, anti-extractable nuclear antigen, nuclear antigen, and myelin basic protein. Similarly, the mRNA vaccine codifying the same viral protein may uncover autoimmune diseases in predisposed patients [89]. These cases support the notion of COVID-19 vaccine-triggered autoimmune phenomena.

Table 4 described previous studies regarding vaccine-related abdominal complications.

**Table 4:** Previous case-report about vaccine-related abdominal complications

Authors	Abdominal complications	Type of vaccine	Number of patients described
Scott et al	Gastroparesis	mRNA	1
Terracina et al	Flare of rheumatoid arthritis	﻿mRNA	1
Obeid et al	﻿Reactivation of IgA vasculitis after	mRNA	1
Rahim et al	IgA nephropathy flare-up	mRNA	1
Salem et al	minimal change disease	mRNA	3
Leclerc et al	minimal change disease	Viral vector	1
Shakoor et al	ANCA-Associated Vasculitis	mRNA	1
Lodato et al	Autoimmune hepatitis	mRNA	1
Bril et al	Autoimmune hepatitis	mRNA	1

### Imaging

The possible abdominal manifestations and imaging features of abdominal post-vaccination complications are wide. However, to our knowledge, serious abdominal complications following COVID-19 vaccination could be categorized into two major categories: abdominal vascular complications and vaccine-triggered autoimmune phenomena. With regard to the first category, we discussed the potential imaging strategies in the previous section in accordance with published guidelines, that suggest the use of intravenous contrast-enhanced CT of the abdomen and pelvis for diagnosing vessel thrombosis and organ complications, in patients with “possible” or “probable” vaccine-induced immune thrombocytopenia [72, 75]. On the other hand, if vaccine-induced immune thrombocytopenia is “unlike”, abdominal imaging should be obtained if clinically appropriated [72, 75].

Vaccine-triggered abdominal autoimmune phenomena are presented with a wide spectrum of manifestations, from glomerular disease to autoimmune hepatitis [79, 80, 82–85, 87, 88]. In view of these potential manifestations, several non-invasive imaging studies should be performed, including abdominal ultrasound, CT, and MRI [79, 80, 82–85, 87, 88].

## Miscellaneous

Among COVID-19 vaccination side effects, some authors described dermatological complications [90–95]. Devon E McMahon reported a spectrum of cutaneous reactions after the mRNA vaccine, such as delayed large local reactions, local injection site reactions, urticarial eruptions, morbilliform eruptions, pernio/chilblains, cosmetic filler reactions, zoster, herpes simplex flares, and pityriasis rosea-like reactions. The authors highlighted that all the skin reactions in the registry are self-limited and minor [92]. Similar results were reported in the literature review by Gronbeck et al., describing that skin reactions were more common following mRNA vaccine and widely self-limited [90].

Another COVID-19 side effect reported was lymphadenopathy [77, 96–99]. In particular, Ozutemiz presented a case series of 5 vulnerable oncologic patients with axillary lymphadenopathy after COVID-19 vaccination [98]. Their results indicated that the lymphadenopathy following immunization may constitute a benign and self-limited condition [98].

Cocco et al. investigated the multiparametric ultrasound findings of patients with post-vaccine lymphadenopathy, describing “worrisome” features, usually suspicious for malignancy including size, shape, morphology, cortex–hilum, SMI, and elastography. The authors highlighted the importance of knowledge of post-vaccination lymph node hypermetabolism, especially in cancer patients to avoid unnecessary biopsy, and appropriately select patients that need a short-term ultrasound follow-up [96].

Previous studies showed potential ocular side effects after COVID-19 vaccination, including panuveitis, acute macular neuroretinopathy, central serous retinopathy [100, 101]. Although no severe ocular complications were described in the registration trials, different vaccines have also been associated with ocular manifestation [100]. In addition, acute corneal graft rejection was described. A case of a 73-years-old man with a penetrating keratoplasty due to keratoconus presented with discomfort in his left eye 13 days after receiving the first dose of mRNA vaccine, with a subsequent diagnosis of corneal graft rejection which improved after drops and oral cortisone [102]. A 66-year-old Caucasian woman endothelial keratoplasty transplant recipient developed acute onset of blurred vision, redness, and photophobia after mRNA vaccine with a clinical appearance typical for acute endothelial graft rejection [103]. Transplant rejection has also been documented in other solid organs [104, 105]. Di Bello et al. reported a case of a 23-year-old who underwent a kidney transplant who presented with acute rejection after the second dose of the mRNA vaccine [104]. Similarly, Vyhmeister et al. described an episode of acute cellular rejection in a liver transplants recipient occurring after the first dose of the mRNA vaccine [105]. In this setting of rapid vaccine deployment, little is known of the efficacy and potential risks of novel SARS-CoV-2 vaccination in transplant recipients, whether this link might be causality or casualty.

Table 5 summarized previous research regarding unusual adverse events after COVID-19 vaccination.

**Table 5:** Previous case-report about vaccine-related miscellaneous complications

Authors	Others complications	Type of vaccine	Number of patients described
Blumenthalet al	Delayed Large Local Reactions	mRNA	12
Johnston et al	Delayed Localized Hypersensitivity Reactions	mRNA	16
Ackerman et al	Persistent maculopapular rash	mRNA	1
Ohsawa et al	Morbilliform rash	mRNA	1
Ozutemiz et al	Lymphadenopathy	mRNA	5
Singh et al	Lymphadenopathy	mRNA	1
Fernández-Prada et al	Lymphadenopathy	mRNA	20
Fowler et al	central serous retinopathy	mRNA	1
Mudie et al	Panuveitis	mRNA	1
Wasser et al	Keratoplasty Rejection	mRNA	2
Phylactou et al	endothelial corneal transplant rejection	mRNA	2
Del Bello et al	Transplant rejection	mRNA	1
Vyhmeister et al	Transplant rejection	mRNA	1

## Conclusion

Although multiorgan adverse events have been reported with the COVID-19 vaccines, the benefits of immunization in preventing severe morbidity and mortality overcomes the risk of vaccinations against SARS-COV-2. However, clinicians should be aware of these potential complications when evaluating and managing patients after COVID-19 vaccination, allowing a prompt diagnosis to improve the patient outcomes.

## Data Availability

The data of this manuscript are available.

## Abbreviations

CCTA: Coronary computed tomography angiography
CDC: Center for Disease Control and Prevention
CMR: Cardiac magnetic resonance
COVID-19: Coronavirus disease 2019
CTA: Computed tomography angiography
MHRA: Medicines and Healthcare Products Regulatory Agency
MRA: Magnetic resonance angiography
SARS: Severe acute respiratory syndrome
STEMI: ST-segment elevation myocardial infarction
VAERS: Vaccine Adverse Event Reporting System
